# Considerations of Mutual Exchange in Prosocial Decision-Making

**DOI:** 10.3389/fpsyg.2019.01216

**Published:** 2019-05-28

**Authors:** Suraiya Allidina, Nathan L. Arbuckle, William A. Cunningham

**Affiliations:** ^1^ Department of Psychology, University of Toronto, Toronto, ON, Canada; ^2^ Department of Psychology, Canisius College, Buffalo, NY, United States

**Keywords:** reciprocity, prosociality, altruism, mutual exchange, social decision-making

## Abstract

Research using economic decision-making tasks has established that direct reciprocity plays a role in prosocial decision-making: people are more likely to help those who have helped them in the past. However, less is known about how considerations of mutual exchange influence decisions even when the other party’s actions are unknown and direct reciprocity is therefore not possible. Using a two-party economic task in which the other’s actions are unknown, Study 1 shows that prosociality critically depends on the potential for mutual exchange; when the other person has no opportunity to help the participant, prosocial behavior is drastically reduced. In Study 2, we find that theories regarding the other person’s intentions influence the degree of prosociality that participants exhibit, even when no opportunity for direct reciprocity exists. Further, beliefs about the other’s intentions are closely related to one’s own motivations in the task. Together, the results support a model in which prosociality depends on both the social conditions for mutual exchange and a mental model of how others will behave within these conditions, which is closely related to knowledge of the self.

Despite initial views of human nature as inherently self-interested, early research in economics and psychology demonstrated that prosocial considerations of other people play a large role in decision-making. Evidence from a wide variety of economic decision-making tasks indicates that people commonly sacrifice personal gains in order to help unknown others. For example, when given the opportunity to divide a sum of money between themselves and an unknown other player, participants consistently give above-zero amounts to the other person, demonstrating a consideration for the outcomes of this unknown other at the expense of their own rewards (e.g., Forsythe et al., 1994; Eckel and Grossman, 1996; Bolton et al., 1998; Engel, 2011; House et al., 2013; Thunström et al., 2016). While a variety of reasons for prosocial giving have been suggested, ranging from moral preferences for doing the right thing (Capraro and Rand, 2018; Tappin and Capraro, 2018) to adherence to social norms (Krupka and Weber, 2013; Kimbrough and Vostroknutov, 2016; Zhao et al., 2017), a key factor influencing decisions seems to be reciprocity. Namely, people are more likely to help someone who has helped them in the past, especially if they can attribute good intentions to this person. Here, we extend research on reciprocity and prosociality by testing whether the mere opportunity for mutual exchange increases prosocial decision-making, even when direct reciprocity is not possible. We further examine how implicit “person models” of the other person relate to the degree of prosociality exhibited within such a mutual exchange.

## Background

Over the past few decades, researchers have debated the causes and mechanisms of prosocial behavior within economic decision-making tasks. Some have proposed that aversion to inequity (Fehr and Schimidt, 1999; Bolton and Ockenfels, 2000) or, more recently, moral preferences for doing the right thing (Capraro and Rand, 2018; Tappin and Capraro, 2018) drive this prosociality. However, a large body of work suggests that these preferences depend partially on the task context, and decisions in these tasks cannot be explained solely by explanations revolving around morality. Rather, research within the past decade has highlighted the importance of social norms in driving prosocial behavior using tasks such as the Dictator Game, in which one participant (the “dictator”) is endowed with a portion of money and must choose how much of that sum to give to another participant (the “recipient”). Contrary to the idea that stable individual differences drive prosociality in these tasks, willingness to give to others is affected by the social appropriateness of the decision (Krupka and Weber, 2013) and by the general tendency of the giver to follow norms (Kimbrough and Vostroknutov, 2016; Zhao et al., 2017). Subtle changes to the design of these economic games, such as manipulating what is considered “fair” (List and Cherry, 2008), drastically reduce the amount of money that people choose to give to the recipient, presumably by changing the normative response. For example, expanding the range of options by allowing participants to take money away as well as to give decreases the amount given nearly to zero (List, 2007; Bardsley, 2008), although this does not occur when the same set of options is simply framed as “taking” money from someone rather than “giving” it to them (Dreber et al., 2013; Goerg et al., 2017; Capraro and Vanzo, in press). Furthermore, when the participant’s accountability to social norms is decreased, either through increased anonymity (Franzen and Pointner, 2012) or increased uncertainty about the relationship between decisions and outcomes (Dana et al., 2007), the average amount given shows a large decrease. The amount given by both children and adults also varies across different cultures, each with their respective norms regarding prosociality (House et al., 2013; House, 2016). Together, these results point to adherence to social norms as a main motive for prosociality in economic tasks.

One powerful norm within interpersonal exchanges is reciprocity, the idea that people will repay prosociality from another by acting prosocially in return (Trivers, 1971; Falk and Fischbacher, 2006; Moreno-Okuno and Mosiño, 2017). A large body of research using economic games has provided evidence for direct reciprocity, with people choosing to give more to a recipient when the recipient has previously helped them (e.g., Ben-Ner et al., 2004; Leiberg et al., 2011; Krockow et al., 2016). For example, when the recipient and dictator in an initial Dictator Game switch roles and play a second time, the amount of money given in the second game is strongly correlated with the amount received in the first game. This correlation is greatly reduced when the former recipient instead plays with an entirely new player in the second game (Ben-Ner et al., 2004). The literature on “conditional cooperation” provides further support for the role of reciprocity, indicating that people are more willing to contribute more to a public good if they know that others are also contributing more (Fischbacher et al., 2001; Frey and Meier, 2004; Keser and van Winden, 2006). Finally, reciprocity has also been conceptualized as an internalized social norm (Perugini et al., 2003), with individual differences in the tendency to reciprocate predicting behavior within common economic games (Li et al., 2017). Importantly, considerations of reciprocity in many of these examples are not strategic, as no additional money can later be gained from partners through promises of reciprocity.

Reciprocal giving in economic exchanges critically depends on attributing positive intentions to the other person (Falk et al., 2008): not only must the actions of a partner be prosocial, one must also be able to infer good intentions behind these actions in order for reciprocity to occur. When the other party’s actions are observable and decoupled from outcomes, reciprocity is based largely off of intentions (Charness and Levine, 2007; Rand et al., 2015), though outcomes sometimes affect decisions as well (Cushman et al., 2009; Schächtele et al., 2011). Indeed, people choose to reciprocate more when good intentions can be attributed to the giver (Falk and Fischbacher, 2006), either through the knowledge that the giver chose to help rather than helping involuntarily (Greenberg and Frisch, 1972; McCabe et al., 2003) or through ruling out strategic motivations for the giver’s behavior (Stanca et al., 2009). Even young children infer the motivations of others when deciding whether to help them, refusing to help those who have harmful intentions toward others (Vaish et al., 2016).

## Current Research

Considerations of reciprocity clearly influence prosocial decision-making in response to another’s actions; however, it is unclear whether considerations of mutual exchange can affect such behavior even when no knowledge of the other person’s actions is available. Rather than choosing whether to reciprocate another’s helpful or hurtful actions, we are often faced with the opportunity to act prosocially toward someone whom we have no prior experience with. Will we be more likely to help this person if we know that their choices are simultaneously affecting our own outcomes? In such situations, uncertainty about the recipient’s potential actions toward us may affect how we choose to treat them (Shafir and Tversky, 1992). Here, we build on the vast literature on reciprocity to test whether the mere opportunity for mutual exchange drives prosociality even when direct reciprocity is not possible. Further, we test whether one’s beliefs about a partner’s intentions influence the degree of prosociality exhibited in a mutual monetary exchange. As each player makes decisions without knowing about the partner’s choices, direct reciprocity in response to the partner’s actions is not possible. Despite this, if people desire mutual exchange with a partner even independently of any potential increases in monetary reward, at least two factors will contribute to prosociality here: an environmental structure that allows for mutual exchange between people (Study 1), and the belief that the other party in the exchange has good intentions toward you (Study 2).

In situations where one has no knowledge of the other player, inferring the other’s intentions requires the application of prior beliefs, or “person models” (Park et al., 1994; Mohr and Kenny, 2006), of what others are like in general. Different people bring different prior beliefs to the game: one participant may believe that people generally help others when they have the opportunity to do so, while another may believe that most people are selfish and consider only themselves. Research on social projection theory suggests that these person models are at least partially acquired through projection of one’s own intentions and motivations: people believe others are similar to them (Krueger, 2007; Acevedo and Krueger, 2011; Krueger et al., 2012). Thus, prior beliefs about others’ motivations and actions may mirror how an individual makes her own decisions.

In order to examine the role of mutual exchange and inferred intentions in acts of prosociality, we conducted two studies using the dual gamble task (Arbuckle and Cunningham, 2012). In this task, participants are presented with a series of paired gambles, one for the self and one for an unknown other participant. On each trial, each player must choose whether to take or pass the pair of gambles that is presented; they must take or pass both gambles together. Outcomes are revealed for gambles that are taken, and these outcomes are added or subtracted to the participant’s total score.

The dual gamble task allows for cooperation to be mutually beneficial – if both players are prosocial and choose to help the other even at a loss to themselves, each player can do much better than if both players were fully selfish. Critically, however, even players who are not willing to take on significant losses for themselves can still act prosocially in this task. In particular, they can choose to consider the other’s gambles only when the expected value of their own gambles is close to 0. Thus, whereas in traditional economic games selfishness and concern for others are perfectly negatively correlated (e.g., giving to another in the Dictator Game means losing money for yourself), the dual gamble task provides a way for each person to simultaneously affect the other’s outcomes without the necessity for self-sacrifice (see Supplementary Table 1 for details).

The first study was designed to examine whether prosociality relies on a task structure that allows for mutual exchange, even when direct reciprocity is not possible. To do this, we manipulated whether the other player’s decisions affected the participant’s outcomes in the task. In Study 2, we examined the relationship between prosociality toward another and beliefs about that other’s actions and intentions, again when there is no opportunity for direct reciprocity. Together, these two studies test the proposal that prosociality toward an unknown other whose actions are unobservable depends on both environmental conditions that allow for exchange and beliefs that the other has good intentions toward you.

Although classic economic games like the Dictator Game have demonstrated that people do give above-zero amounts to another person even when the other has no opportunity to reciprocate, subsequent research has suggested that this type of prosocial giving is driven largely by perceived norms surrounding the amount that should be given (Krupka and Weber, 2013; Kimbrough and Vostroknutov, 2016). Indeed, when steps are taken to reduce such norms, the amounts given are reduced almost to zero (Dana et al., 2007; List, 2007; Bardsley, 2008; List and Cherry, 2008; Franzen and Pointner, 2012). Since the dual gamble task involves trial-by-trial probabilistic outcomes rather than a one-shot deterministic outcome, the participant’s weighting of self and other outcomes is much less evident than in the Dictator Game, potentially reducing the influence of perceived norms. Furthermore, doing well for the self in the dual gamble task requires more effort than in the Dictator Game, as on each trial participants must integrate information about the probability of the gamble winning and the amounts that will be won or lost when the gamble is played. This increased effort may reduce the attention placed on perceived norms. Thus, prosociality in the no-reciprocity condition may be reduced compared to past research using the Dictator Game.

## Study 1

To test whether mutual exchange is necessary for prosociality in this task, we manipulated whether the participant’s final outcomes were affected by the other player’s decisions. To do so, we created two conditions in which only the instructions of the task differed. In the first condition (the Mutual Exchange condition), participants were told that they would be making decisions for themselves and for an unknown other, and that the other player’s decisions would similarly affect them. In the second condition (the One-Way condition), participants were told that their decisions would affect themselves and the other player, but the other player’s decisions would have no effect on them. In this condition, no intentions (positive or negative) can be attributed to the partner, as their decisions do not affect the participant’s outcomes at all.

If participants’ decisions are driven by a desire for fair, mutual exchange, they should only care about helping the other person in the Mutual Exchange condition. If, on the other hand, decisions are driven more by stable other-regarding preferences, participants should care about helping the other player in both conditions, regardless of whether mutual exchange is possible. Importantly, prosociality in this task does not necessarily require self-sacrifice, as participants can choose to consider information about the other only when the expected value for the self-gamble is around 0. Further, any prosociality that is present in either condition is not strategic: even in the Mutual Exchange condition, choosing to help the other has no effect on how the other chooses to play for you, as they have no knowledge of your decisions until after the task is complete.

### Methods

#### Participants

A total of 100 participants (64 women) were recruited from the University of Toronto Rotman School of Management and participated in exchange for research course credit. For each study, no data were analyzed until after all data collection was completed. Participants received a bonus of between $0 and $10 based on the final task outcomes. This research was approved by the University of Toronto Research Ethics Board (protocol #30952). For both studies, written informed consent was obtained from all participants before the study began.

#### Procedure

Participants first read a series of instruction pages explaining the nature of the task. All participants were told that they would be making decisions about whether to take or pass gambles. They were informed that each gamble had a probability of winning and a value associated with it (e.g., 60% chance of winning 7 and 40% chance of losing 1), which would vary from trial to trial. They were to make decisions on whether to take or pass the gambles based on these probabilities and values. Participants were told that they would be presented with two gambles at a time, one for the self and one for an unknown other participant who they were paired with. On each trial, they would have to decide whether to take or pass the gambles; they could not choose between gambles but had to either take or pass both gambles together. Gambles that they chose to take would be played and the outcome added to their total points, while gambles that they chose to pass would not.

Participants were randomly assigned to one of two conditions, in which only the instructions of the task differed. In the Mutual Exchange condition (*N* = 49), participants were told that each player’s final payment would depend on both their own decisions and the other player’s decisions. Specifically, participants’ final payments would depend on the total number of points that they got for themselves and the total number of points that the other player got for them. Similarly, the partners’ final payments would depend on the number of points that participants got for them and the number of points that they got for themselves. In the One-Way condition (*N* = 51), participants were told that their own payment would depend only on the points that they got for themselves; the other player’s decisions would have no impact on them. However, the decisions that the participant made for the other would still affect the total payment received by the partner, as in the first condition.

All participants were then presented with pairs of gambles and made decisions to take or pass these gambles. Each participant completed a total of 80 trials. The probability and value of each gamble varied from trial to trial, with possible probabilities of 20, 40, 60, and 80%, possible gains of +1, +4, +7, and +10, and possible losses of −10, −7, −4, and −1. Probabilities and values were selected randomly and independently on each trial. No deception was used in the study; participants in the Mutual Exchange condition actually received money proportional to the points that they got for themselves and the points the other got for them, while those in the One-Way condition received money based only on their own decisions in the task. All manipulations, analyzed measures, and exclusions in both studies are disclosed here.

### Results

#### Gameplay Behavior

To examine how participants use information about the self and the other when making decisions, we computed “expected value” (EV) variables for the Self and Other gambles as follows: EV = (probability of winning) × (amount of possible points won) − (probability of losing) × (amount of possible points lost). We then modeled decisions (take or pass) with multilevel logistic regression (using glmer from the “lme4” package in R) as a function of the expected value for the Self gamble (SelfEV), the expected value for the Other gamble (OtherEV), and the interaction between these variables (SelfEV × OtherEV), with trials nested within participants. Replicating previous work (Arbuckle and Cunningham, 2012), we found positive effects of SelfEV (*β* = 0.463, *p* < 0.001) and OtherEV (*β* = 0.034, *p* < 0.001) on the decision to take a gamble, indicating that on average, people make good decisions for themselves and others. The effect of SelfEV was larger than that of OtherEV, *χ* ^2^(1) = 974.48, *p* < 0.001, indicating that one’s own outcomes are given higher importance than others’ outcomes. The significant interaction between SelfEV and OtherEV on the decision to take a gamble (*β* = 0.008, *p* = 0.012) suggests that participants take OtherEV into account slightly more when SelfEV is not negative.

#### Modeling the Influence of the Potential for Mutual Exchange

The main purpose of this study was to examine the motivations underlying prosocial behavior by manipulating whether the participant’s outcomes are dependent on the other player. The critical question was whether the use of self and other information would change when there is no opportunity for mutual exchange (i.e., when the other player’s decisions do not affect the participant’s outcomes). To test this, we created a model predicting decision from SelfEV, OtherEV, Condition (Mutual Exchange or One Way), and the interaction of all variables.

**Figure 1:**
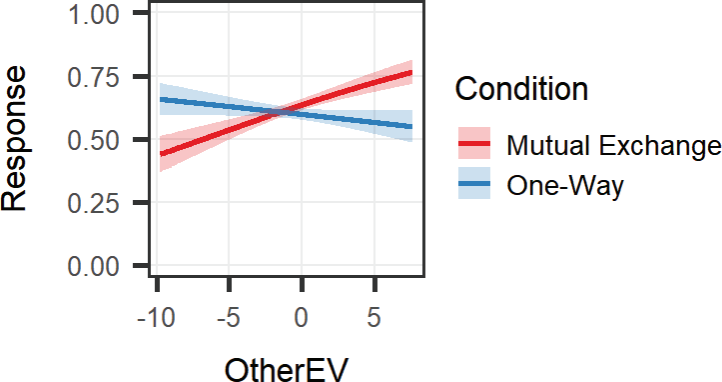
Interaction of Condition with OtherEV on the decision to take a gamble. Participants in the Mutual Exchange condition were more likely to take gambles that were good for the other and pass on gambles that were bad for the other, while those in the One-Way condition did not use OtherEV when making decisions.

Two different patterns of results are possible, leading to potentially different conclusions about the motivations underlying reciprocity. If people help others in this task simply due to a desire to benefit the other person, prosociality should be present in both the Mutual Exchange condition and the One-Way condition. If, on the other hand, people assume that others are prosocial and therefore help others out of a desire for fairness and mutual exchange, prosociality should only be present in the Mutual Exchange condition, in which each person’s decisions affect the other. In line with the latter possibility, the results indicate that participants made worse decisions for the other in the One-Way condition compared to the Mutual Exchange condition (see Table 1 for estimates and *p*’s). When the other player’s decisions did not affect their outcomes, participants no longer made prosocial decisions, even when they could do so at no cost to themselves (see Figure 1). This suggests that the mutual exchange present when each player’s outcomes depend on the other is necessary for prosociality to emerge in this task. A three-way interaction between SelfEV, OtherEV, and Condition was also significant, suggesting that the differences in the use of OtherEV between conditions are the greatest when SelfEV is around 0 (see Supplementary Figures 1, 2).

**Table 1:** Estimates and *p*’s, Study 1.

Term	Beta	*p*
SelfEV	0.591	0.001***
OtherEV	0.088	0.004**
Condition	−0.137	0.325
SelfEV:OtherEV	0.016	0.012*
SelfEV:Condition	0.064	0.362
OtherEV:Condition	−0.115	0.007**
SelfEV:OtherEV:Condition	−0.017	0.040*

*Results from the model predicting choice (take or pass) from SelfEV, OtherEV, Condition, and the interaction of all variables (Study 1)*.

* <0.05;

** <0.01;

*** *<0.001*.

### Summary

Study 1 revealed that participants made prosocial decisions in the dual gamble task only when they believe that each player’s decisions are affecting the other’s outcomes, creating mutual dependence. When this exchange is not present, people no longer take the other’s outcomes into consideration when making decisions, even when they can do so without harming their own outcomes. These results suggest that prosociality in this task is driven by a desire for fair and mutual exchange rather than simply by altruistic motivations to help the other person.

Participants in the Mutual Exchange condition may help others because they believe that others will help them in return, such that both players will be better off than if each had acted selfishly. Those in the One-Way condition know that the other cannot help them, and that to maximize self-benefit they therefore must focus on helping themselves. Critically, however, even when these participants can help the other person without hurting their own outcomes (i.e., by considering the OtherEV only when the SelfEV is around 0), those in the One-Way condition still do not act prosocially. Thus, it seems that in situations where mutual exchange is impossible, people do not help others even when there is no cost to doing so.

## Study 2

In Study 1, we demonstrated that prosocial behavior is contingent on the potential for mutual exchange; people only act prosocially if the target of their prosociality is simultaneously affecting the participants’ outcomes. The necessity of mutual exchange for prosociality suggests that participants believed that the other player would help them when given the chance, and they therefore helped in return. However, while participants on average may have believed the other was prosocial, the degree to which individuals help others may depend on individual differences in beliefs about what the other person is likely to do. Someone who believes that the other player will help them is likely to help in return, as seen in Study 1, whereas someone who believes that the other will act selfishly may no longer be prosocial. Thus, in Study 2 we examine how beliefs about the other player’s likely motivations and actions relate to participants’ behavior.

To test whether prosociality in this task depends on an individual’s implicit “person model” of what the other is likely to do, we assessed participants’ beliefs about the other player’s motivations and behaviors. Participants completed the standard dual gamble task, which was identical to the Mutual Exchange condition of Study 1. They then rated their agreement with a series of statements about their own gameplay (how well they think they did for themselves and for the other), their motivations during the task (how much they care about doing well for the other and about outperforming the other), and their estimations of the other’s gameplay and motivations (how well the other person did for them and how much the other person cares about doing well for them).

### Methods

#### Participants

A total of 122 US-based participants were recruited from Amazon Mechanical Turk (AMT) for payment. Using AMT ensures increased anonymity, as there is no social interaction either between participants or between experimenters and participants. Two participants were excluded from analysis for lack of variation in responses (one participant passed on 100% of trials, one participant took on 97% of trials), leaving 120 total participants for analysis (66 women). Participants were given 50 cents base pay upon 48 h of completing the study, and within 2 weeks were given bonus pay between $0.50 and $5.00, based on the decisions they made for themselves and the decisions that the other person made for them.

#### Procedure

Participants completed the standard dual gamble task, which was identical to the Mutual Exchange condition in Study 1 except that each participant completed 100 trials. After completing all the trials, participants were asked 11 questions about their own gameplay (e.g., how well they think they did for themselves and for the other player), their motivations during the task (e.g., how much they care about how well they did for the other person and how much they want to outperform the other person), and their estimations of the other’s gameplay and motivations (e.g., how well the other person did for them and how much the other person cares about them).

### Results

#### Motivations for Gameplay Behavior

Although participants on average reported prosocial intentions and expectations for gameplay, there was substantial variability in these reports (means and standard deviations are presented in Supplementary Table 2). Examining the correlations among these variables provides support for the idea that expectations for the other person’s intents and gameplay are related to one’s own gameplay. Participants who reported that they cared about the other player indicated that the other player cared about them, *r*(118) = 0.73, *p* < 0.001. In addition, those who believed that the other player did well for them reported that they did well for the other, *r*(118) = 0.44, *p* < 0.001. Similarly, the number of points that participants estimated they got for the other correlated with the points they estimated the other got for them, *r*(118) = 0.87, *p* < 0.001.

To create composite scores for further analysis, we conducted a factor analysis of the items that asked about expected outcomes for the self and other and motivations for these behaviors. This analysis indicated that two factors could account for 48.8% of the variance. Factor loadings were subjected to a promax rotation to allow for correlation between the factors (see Supplementary Table 3 for factor loadings). Examining the structure of these factors indicated that participants conceptualized the actual outcomes and performance separately from the motivations for attending to self and other information. As was suggested by the correlations noted above, caring for the other was highly linked to how much one thought that the other cared for them. These results suggested that we could create two composite scores by aggregating three items assessing participants’ prosocial motivations toward the other player and estimations of the other’s motivations toward them, and then aggregating three items assessing how well participants did for themselves and for the other and how well they believed the other did for them (results of the following models do not substantially change if we exclude the item assessing how well participants did for themselves, which had a factor loading of only 0.318, from the latter variable). We labeled these two variables Motivations and Outcomes, respectively. The final item, assessing how important it is for the participant to get more points than the other player, loaded on both factors and therefore was not included in either variable.

#### Gameplay Behavior

To examine how participants use information about the self and the other when making decisions, we modeled decisions as a function of SelfEV, OtherEV, and the interaction between these variables, with trials nested within participants. Replicating Study 1, this analysis revealed positive effects of SelfEV (*β* = 0.479, *p* < 0.001) and OtherEV (*β* = 0.092, *p* < 0.001) on the decision to take a gamble, indicating that participants take the expected value for both self and other into account. As in Study 1, the effect of SelfEV on decisions was larger than that of OtherEV, indicating that concern for one’s own outcomes outweighs concern for others’ outcomes. The interaction between SelfEV and OtherEV was not significant (*β* = 0.003, *p* = 0.235).

A critical question for this study was how beliefs about the other player and their motivations would influence how self and other information was used during gameplay. To examine these questions, we ran a series of models to test whether each individual difference variable moderates the basic effects of SelfEV and OtherEV. Results from the individual models are reported here, but these results are similar when all three individual differences are run in the same model.

##### Modeling Motivations

**Figure 2:**
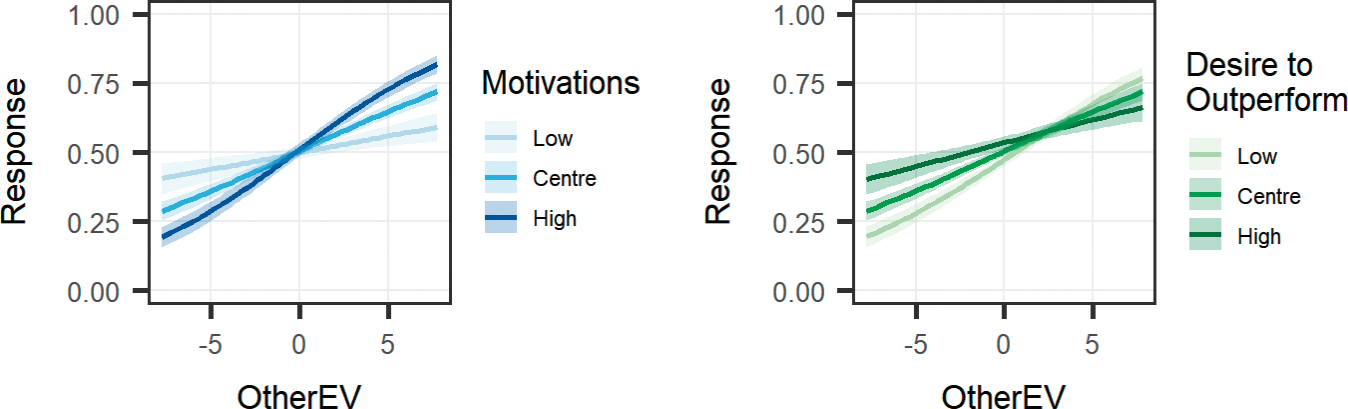
Interaction of Motivations and Desire to Outperform with OtherEV on the decision to take a gamble. Participants who scored high on Motivations were more likely to make good decisions for the other, while those who scored high on Desire to Outperform were less likely to make good decisions for the other.

If inferring the other player’s intentions drives prosociality in this task, we would expect scores on the Motivations variable to interact with the use of OtherEV when making decisions. To test this, we created a model predicting decision from SelfEV, OtherEV, Motivations, and the interaction of all variables. Consistent with this hypothesis, the results indicated that Motivations interacted with OtherEV (*β* = 0.070, *p* < 0.001), such that those who reported higher estimations of their own and the other’s prosocial motivations were more likely to use the other information when making decisions (see Figure 2). No interaction was found between Motivations and SelfEV (*β* = −0.042, *p* = 0.194), indicating that other-related motivations did not affect participants’ use of self-relevant information when making decisions.

##### Modeling Expected Outcomes

To examine how estimations of each player’s outcomes moderate the use of self and other information, we created a model predicting decision from SelfEV, OtherEV, Outcomes, and the interaction of all variables. This analysis indicated that Outcomes did not interact with SelfEV (*β* = 0.003, *p* = 0.928) and only marginally interacted with OtherEV (*β* = 0.033, *p* = 0.084), such that those who reported higher outcomes for self and other and higher estimations of the other’s outcomes for them were slightly more likely to take the Other information into account when making decisions.

##### Modeling the Desire to Outperform

We then examined how the desire to outperform the other player moderated the use of self and other information in this task. We created a model predicting decision from SelfEV, OtherEV, desire to outperform, and the interaction of all variables. The results reveal that those who wish to outperform the other were less likely to use the OtherEV when making decisions, (*β* = −0.050, *p* = 0.008; see Figure 2). Desire to outperform did not interact with SelfEV, (*β* = −0.040, *p* = 0.212). A three-way interaction was found between SelfEV, OtherEV, and the desire to outperform (*β* = −0.007, *p* = 0.040; see Supplementary Figure 3).

To examine the simple slopes of this three-way interaction, we re-centered SelfEV and OtherEV at three different levels (1.5 SD below the mean, at the mean, and 1.5 SD above the mean) and ran a series of models predicting choice from each level of SelfEV and OtherEV, with desire to outperform as a moderator (Aiken and West, 1991). The desire to outperform the other player predicted taking gambles that were both bad for the other player and bad or neutral for the self (see Supplementary Table 4 for estimates). This suggests that those who strongly desired to outperform the other player were willing to hurt themselves in order to hurt the other.

##### Modeling Estimated Total Points

To further examine the factors shaping how participants make decisions in this task, we examined how their estimations of the total points gained by each person related to their use of self- and other-related information during the task. We created a model predicting decision from SelfEV, OtherEV, estimated points gained for the self, estimated points gained for the other, and estimated points the other gained for you (estimates are reported in Supplementary Table 5). Participants’ estimates of how many points they got for themselves predicted better decisions for the self (interaction between estimated points for the self and SelfEV: *β* = 0.153, *p* = 0.038), and their estimates of how many points they got for others predicted worse decisions for themselves (interaction between estimated points for the other and SelfEV: *β* = −0.152, *p* = 0.034). These results suggest that participants’ use of SelfEV (but not OtherEV) relates to their estimates of how they did in the task – those who think they got many points for themselves indeed used SelfEV more, while those who think they got many points for others used SelfEV less.

### Summary

The results of Study 2 reveal that motivations and outcomes are differentiated from each other in the dual gamble task, and that estimations of the other person’s motivations and outcomes closely relate to beliefs about one’s own motivations and outcomes. Motivations, not outcomes, predicted making better choices for the other, while the desire to outperform the other predicted worse choices for the other even if doing so meant harming oneself. This suggests that inferences about the other player’s intentions in this task are key to prosociality, influencing prosocial decision-making even when the other’s actions are unknown.

## Discussion

The results of two studies indicate that both the conditions for mutual exchange (Study 1) and the belief that the other will act prosocially within those conditions (Study 2) are necessary for prosociality in this task to arise. Study 1 demonstrated that people only help others when they believe that others have the opportunity to help them in return, indicating that conditions for mutual exchange are vital for prosociality. Study 2 showed that inferences about the other player’s motivations and outcomes are closely tied to estimates of one’s own motivations and outcomes, with intentions rather than perceived outcomes driving prosociality in the task. Together, these studies suggest that prosociality hinges on both the necessary social dynamics for reciprocity and a mental model of others’ intentions within these social dynamics.

Contrasting previous research using the Dictator Game, Study 1 found that people do not act prosocially when their partner has no reciprocal influence on their outcomes. As prosocial behavior in some economic tasks may occur out of the desire to avoid appearing unfair to oneself or to others (Dana et al., 2007), one explanation for this difference lies in the transparency of a participant’s motivations. While tasks such as the Dictator Game make the participant’s valuation of the self and other extremely clear, these motivations cannot be as readily inferred from outcomes in our task. Since the dual gamble task requires participants to incorporate multiple pieces of information (i.e., the probabilities, win values, and loss values for both the self and other gambles) and simultaneously allows a large role for randomness, self-presentation effects may play less of a role here than in the Dictator Game. Thus, when the desires to appear prosocial to oneself, to the recipient, and to the experimenter have less of an influence, prosocial considerations are greatly reduced. This fits with research demonstrating that giving in the Dictator Game is drastically reduced when accountability is decreased through increased anonymity (Franzen and Pointner, 2012) or increased uncertainty about the relationship between decisions and outcomes (Dana et al., 2007).

When mutual exchange is possible, participants must infer the intentions of the other person to form a model of how they will behave. When faced with a lack of alternative sources of information about the other player, our findings suggest that participants’ inferred mental models of the other tend to closely mirror their own intentions. This mirroring may reflect a desire to act prosocially toward those that we perceive as being similarly prosocial. Alternatively, it may be the result of social projection (Krueger, 2007; Acevedo and Krueger, 2011; Krueger et al., 2012), in which participants use their knowledge of their own intentions to infer others’ motivations. Regardless of the direction of causality, these intentions relate to participants’ willingness to make prosocial decisions for the other person. Thus, our results suggest that people ascribe intentions to others that are in line with their own motivations, with these inferred intentions (rather than any inferred outcomes) driving prosociality.

The importance of inferred intentions rather than outcomes in shaping prosociality precludes the idea that fairness- or equity-based motivations drive prosocial giving (as outcomes, not intentions, would be the best indicator of equity). Rather, one possibility is that emphases on intentions are manifestations of a desire for social connectedness, in which those who desire to help others are themselves rewarded in return. Supporting this idea, reciprocity is greater in individuals who are at risk of being socially excluded (Derfler-Rozin et al., 2010), suggesting that mutual exchange can serve as a means of solidifying social connections. Thus, when mutual exchange is possible, choosing to help only those with good intentions toward you may foster trust by contributing to a social structure in which like-minded prosocial people help each other. Of course, prosociality may also occur without the possibility for reciprocal exchange if the potential recipient is in need or the participant feels responsible for their welfare, neither of which was the case in these studies. Instead, we focus here on the conditions necessary for prosociality between two strangers who possess equal resources.

## Data Availability

The datasets generated for this study are available on request to the corresponding author.

## Ethics Statement

This research was approved by the University of Toronto Research Ethics Board (protocol #30952). Written consent was obtained from all participants before the study.

## Author Contributions

NA and WC contributed in original design. NA contributed in data collection. SA, NA, and WC contributed in data analysis, interpretation, and manuscript writing.

### Conflict of Interest Statement

The authors declare that the research was conducted in the absence of any commercial or financial relationships that could be construed as a potential conflict of interest.
