# Supplementary material for: Considerations of Mutual Exchange in Prosocial Decision-Making
Source: Front Psychol. 2019 May 28;10:1216. doi: 10.3389/fpsyg.2019.01216 (PMC6546851; doi:10.3389/fpsyg.2019.01216)
Supplement: Supplementary file 1 [file Table_1.DOCX]

**Supplementary Material**

**Supplementary Table 1. Total expected values for self and other under different game-play strategies.** To illustrate how different strategies in the task affect the final points for the self and other, we calculated the average points that would be obtained across participants under 1) a completely selfish strategy, 2) a prosocial strategy in which people treat themselves and others equally, and 3) a variety of strategies in between in which people favour themselves but also try to help the other^[[1]](#footnote-1)^. These analyses are for illustration purposes only and of course are not an exhaustive list of the strategies people may take. In particular, these analyses do not consider instances where someone adopts a certain strategy under the assumption that others will choose a different strategy.

| Strategy | Total Expected Value for the Self | Total Expected Value for the Other | Total Expected Value from the combination of Self and Other |
| --- | --- | --- | --- |
| Selfish Strategy | 153.3 | 52.9 | 206.2 |
| Prosocial Strategy | 124.2 | 128.3 | 252.5 |
| Combined Strategy  (consider OtherEV only when SelfEV is between -1 and 1) | 148.7 | 81.2 | 229.9 |
| Combined Strategy  (consider OtherEV only when SelfEV is between -2 and 2) | 135.1 | 104.6 | 239.7 |

**Supplementary Table 2. Means and standard deviations, Study 2.** Means and standard deviations of questions posed to participants after completing the dual gamble task in Study 2. Scales for questions 1-8 ranged from 1 (Disagree strongly) to 7 (Agree strongly). Participants indicated a high degree of understanding of the task (question 1), and other question means are around the midpoint of the scale. For questions 9-11, participants entered their estimated number of points into a text box.

| Item | Mean (SD) |
| --- | --- |
| 1. I understood the gambling task. | 6.40 (1.04) |
| 2. I think I did well for myself on this task | 4.90 (1.34) |
| 3. I think I did well for the other person I was paired with on this task | 4.58 (1.03) |
| 4. I think the other person I was paired with did well for me on this task | 4.38 (0.94) |
| 5. I care about how well I did for the other person on this task | 4.79 (1.69) |
| 6. I think the other person I was paired with cares about how well he or she did for me | 3.95 (1.45) |
| 7. It is important to me that I did well for the other person on this task | 4.31 (1.72) |
| 8. It is important to me that I have more points than the other person that I was paired with | 3.48 (1.82) |
| 9. How many points do you think you got for yourself? | 82.89 (84.57) |
| 10. How many points do you think you got for the other person? | 73.01 (77.35) |
| 11. How many points do you think the other person got for you? | 61.91 (67.92) |

**Supplementary Table 3. Factor loadings, Study 2.** A factor analysis was conducted on 7 items asking about participants’ motivations and outcomes during the task and their estimations of the other person’s motivations and outcomes. Factor loadings were subjected to a promax rotation to allow for correlation between the factors. The analysis indicates that items related to participant’s own outcomes and estimations of the other’s outcomes are highly related, and are distinct from items related to participant’s prosocial motivations and estimations of the other’s motivations. The final item, assessing the desire to outperform the other player, loaded similarly on both factors.

| Item | Outcomes | Motivations |
| --- | --- | --- |
| 1. I think I did well for myself on this task |  | 0.317805 |
| 2. I think I did well for the other person I was paired with on this task | 0.281121 | 0.556129 |
| 3. I think the other person I was paired with did well for me on this task |  | 0.723083 |
| 4. I care about how well I did for the other person on this task | 0.892233 |  |
| 5. I think the other person I was paired with cares about how well he or she did for me | 0.789514 | 0.124896 |
| 6. It is important to me that I did well for the other person on this task | 0.830821 |  |
| 7. It is important to me that I have more points than the other person that I was paired with | -0.32436 | 0.405444 |

**Supplementary Table 4. Estimates of desire to outperform the other on choice at re-centred values of expected value for self (SelfEV) and expected value for other (OtherEV), Study 2.** To probe the simple effects of the desire to outperform the other player, we predicted choice at low (-1.5 SD), centre, and high (+1.5 SD) levels of SelfEV and OtherEV, with the desire to outperform the other as a moderator. Main effects of the desire to outperform at these different levels of SelfEV and OtherEV are reported here. Desire to outperform related to the tendency to take gambles that had a low expected value for the other and a low or centre expected value for the self.

|  |  | **OtherEV** | | |
| --- | --- | --- | --- | --- |
|  |  | **Low** | **Centre** | **High** |
| **SelfEV** | **Low** | 0.515* | 0.302 | 0.09 |
|  | **Centre** | 0.312** | 0.099 | -0.113 |
|  | **High** | 0.109 | -0.104 | -0.317 |

**Supplementary Table 5. Estimates of guessing points model, Study 2.** Results from a single model predicting choice (take or pass) from SelfEV, OtherEV, estimated points gained for the self, estimated points gained for the other, estimated points the other gained for you, and the interactions of SelfEV and OtherEV with each guess. The results indicate that estimated points for the self positively relates to use of SelfEV when making decisions, while estimated points for the other negatively relates to use of SelfEV in making decisions. Estimated points that the other got for you positively interacts with both SelfEV and OtherEV to influence decision.

| Item | SelfEV*Item | OtherEV*Item | SelfEV*OtherEV*Item |
| --- | --- | --- | --- |
| How many points do you think you got for yourself? | 0.153* | -0.075^†^ | -0.004 |
| How many points do you think you got for the other person? | -0.152* | 0.009 | -0.006 |
| How many points do you think the other person got for you? | 0.003 | 0.071^†^ | 0.016* |

* < .05, ** < .01, *** <.001, ^†^ <.10


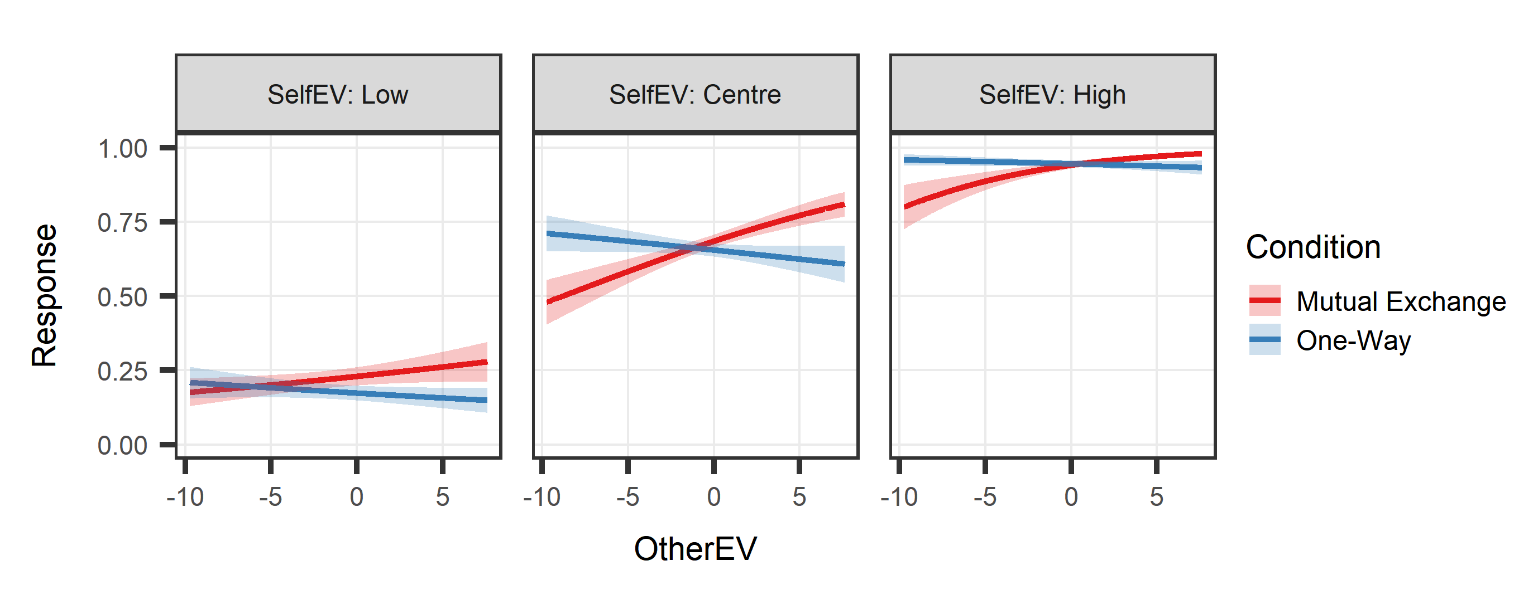
 **Supplementary Figure 1. Three-way interaction between expected value for self (SelfEV), expected value for other (OtherEV), and Condition (Study 1).**

**
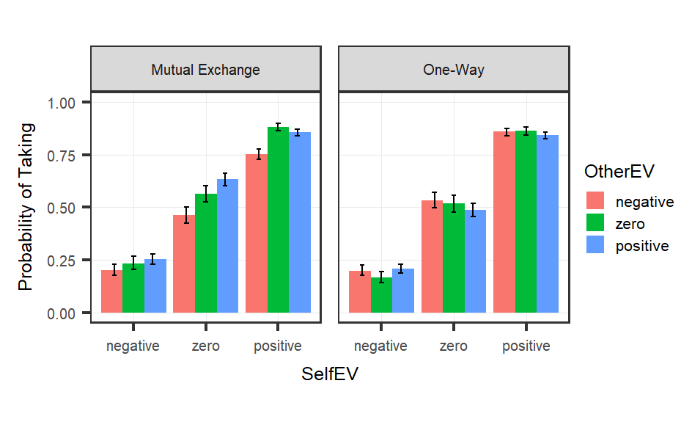
Supplementary Figure 2. Probability of taking the gamble in each condition (Mutual Exchange vs. One-Way) at different levels of SelfEV and OtherEV (where expected values between -1 and +1 are treated as zero; Study 1).**


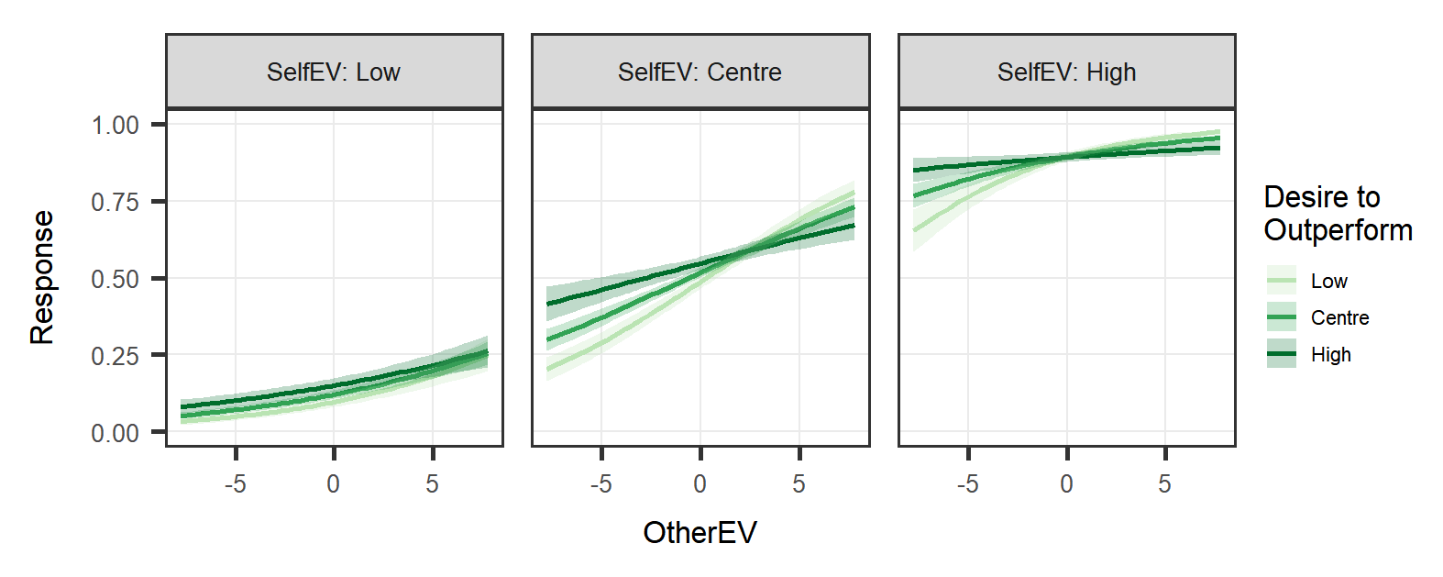
**Supplementary Figure 3. Three-way interaction between SelfEV, OtherEV, and desire to outperform the other player (Study 2).**

1. We thank an anonymous reviewer for suggesting this analysis. [↑](#footnote-ref-1)
